# Implications for directionality of nanoscale forces in bacterial attachment

**DOI:** 10.1007/s41048-016-0019-2

**Published:** 2016-02-22

**Authors:** Jan J. T. M. Swartjes, Deepak H. Veeregowda

**Affiliations:** University of Groningen and University Medical Center Groningen, Department of Biomedical Engineering, Antonius Deusinglaan 1, 9713 AV Groningen, the Netherlands; Ducom Instruments Europe B.V, Center for Innovation, 9713 GX Groningen, the Netherlands

**Keywords:** Bacteria, Bacterial adhesion, Friction, Anisotropy, Shear

## Abstract

Adhesion and friction are closely related and play a predominant role in many natural processes. From the wall-clinging feet of the gecko to bacteria forming a biofilm, in many cases adhesion is a necessity to survive. The direction in which forces are applied has shown to influence the bond strength of certain systems tremendously and can mean the difference between adhesion and detachment. The spatula present on the extension of the feet of the gecko can either attach or detach, based on the angle at which they are loaded. Certain proteins are known to unfold at different loads, depending on the direction at which the load is applied and some bacteria have specific receptors which increase their bond strength in the presence of shear. Bacteria adhere to any man-made surface despite the presence of shear forces due to running fluids, air flow, and other causes. In bacterial adhesion research, however, adhesion forces are predominantly measured perpendicularly to surfaces, whereas other directions are often neglected. The angle of shear forces acting on bacteria or biofilms will not be at a 90° angle, as shear induced by flow is often along the surface. Measuring at different angles or even lateral to the surface will give a more complete overview of the adhesion forces and mechanism, perhaps even resulting in alternative means to discourage bacterial adhesion or promote removal.

Both friction and adhesion play a key role in many natural phenomena. Along with the important role in all kinds of processes, the notion that both friction and adhesion can depend on the applied direction and angle, has intrigued scientists. One well-known example is the occurrence of high and low friction and adhesion cycles in the attachment and detachment of the gecko toe (Tian et al. 2006). Containing millions of small extensions, called spatula, all exerting nanoscale forces to the surface, the gecko can climb even upside down. By rolling its toe, the gecko changes the angle between its spatula and the surface, allowing it to shift between increasing the normal adhesion force and the frictional component (Autumn et al. 2006). At a molecular level, these changes in the angle of the spatula influence the Van der Waals forces in such a way that the attractive force between the spatula and the surface is altered to switch between high and low values (Tian et al. 2006). Simplified, by changing the direction of loading, either the normal adhesion force is high and the friction is low, or the frictional component is high and the normal adhesion force is low.

Whereas geckos can actively choose the loading angle, allowing them to either stay attached or detached, less autonomous systems like molecules and proteins do not have this option. Nevertheless, these systems display forces that highly depend on direction as well. The E2lip3 protein, for example, which is high in beta-sheet content, displays a resistance to pulling that strongly depends on the angle of the applied force (Brockwell et al. 2003). Similar behavior is found in the unfolding of Ubiquitin by mechanical stretching (Carrion-Vazquez et al. 2003). The direction of the applied force determines to a large extent the protein’s stability. In these cases, the different angles in which the force is applied are believed to cause a change in the way the hydrogen bonds of inner beta-sheets rupture. As in the case of the gecko, the angle of the force determines whether bonds are broken by shearing or peeling (Brockwell et al. 2003). The regulation of bond organization by mechanical force has been simplified by describing it either as parallel distribution of forces, where each bond aids in resisting a mechanical force, or a zipper-like distribution (Fig. 1) in which one bond after another is required to oppose detachment (Albrecht et al. 2003; Hess 2006; Isabey et al. 2013). Based on the organization of the bonds, changing the loading direction will shift the distribution of forces, switching from parallel to zipper-like, or the other way around. In the parallel scenario, the collective bond behavior is able to resist much larger forces as the contribution of each bond is added up. A popular example of this is Velcro, which is easily loosened when pulled up from one side, but displays a highly increased resistance to detachment when pulled sideways (Matouschek and Bustamante 2003).

**Fig. 1:**
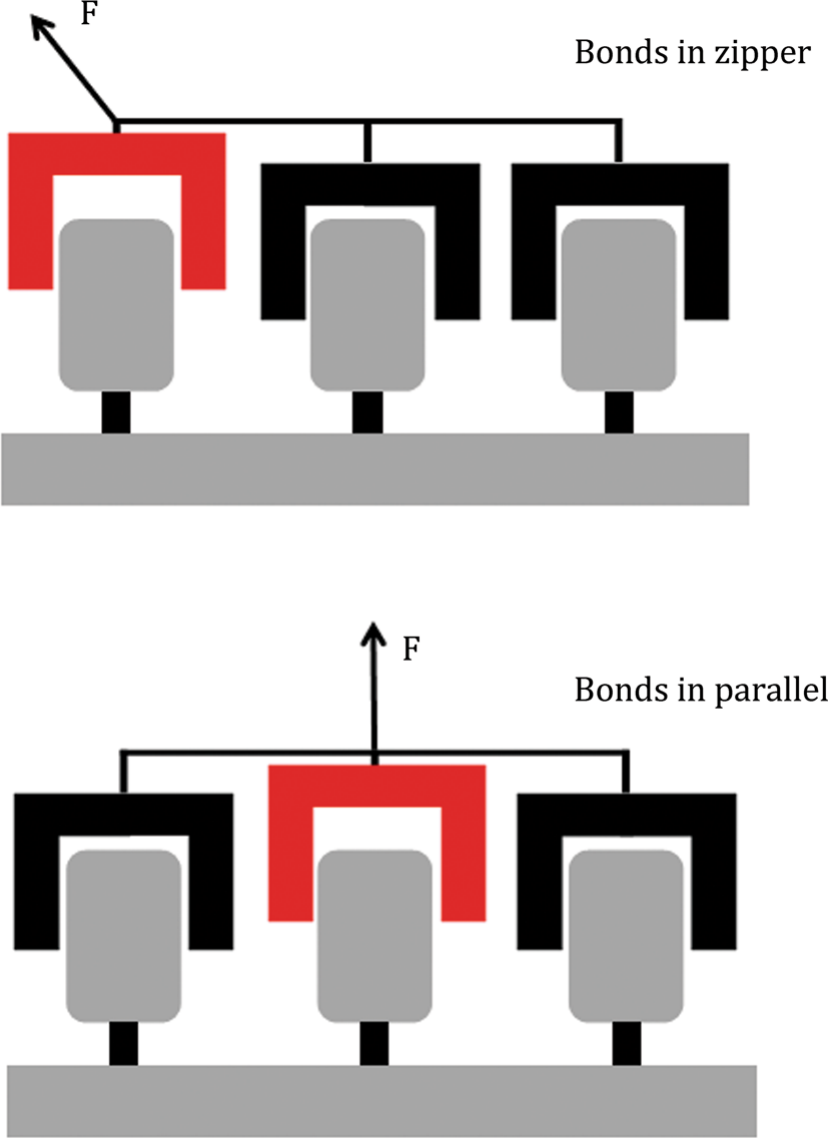
Distribution of forces over multiple bonds. In the zipper-like distribution (*top*) each bond is loaded consecutively, passing the load on to the next bond after breaking. While in the parallel distribution (*bottom*) the load is distributed over all included bonds and after breaking of one bond, the load is redistributed over the remaining ones. Adapted with permissions from Isabey et al. (2013)

Unzipping of proteins is, for example, also observed in the amyloid-like interactions within clusters of adhesins called Als, which contribute in the adhesion of *Candida albicans* (Alsteens et al. 2010, 2012). Force analysis of the adhesion shows mechanical unzipping of β-sheet interactions between Als proteins upon being pulled from an amyloid coated surface at 90°. One step beyond the scale of inter-protein bindings, shear, or lateral, force dependency of adhesion is observed in ligand-receptor complexes. The FimH adhesin expressed by *Escherichia coli* binds to mannose and is found to enhance adhesion under high shear conditions (Thomas et al. 2004; Aprikian et al. 2007). Whereas the behavior of the previously mentioned proteins is generally ascribed to bond organization, it has been suggested that in the case of *E. coli* the typical behavior stems from allosteric regulation, causing bond enhancement by mechanical force (Yakovenko et al. 2008).

From animals to proteins it is clear that the direction of an applied force can make the difference between adhesion or detachment, structural integrity or unfolding. For certain microorganisms, like bacteria, surface attachment is the preferred mode of survival, as stable surface bound communities offer protection to antibiotics and mechanical removal (O’Toole et al. 2000; Dunne 2002; Vlamakis et al. 2013). At the same time, whether the surface comprises the inner-lining of the human body, or an implant, the formation of these biofilm communities is often highly unwanted (Cegelski et al. 2008; Löfling et al. 2011; Foster et al. 2014). For decades, researchers are trying to deal with bacterial adhesion by an almost endless effort to create non-fouling surfaces which can withstand adhesion of bacteria. On the other hand, there is also a tremendous amount of work put into new strategies of effective removal of adhered bacteria. In both cases, fundamental knowledge of bacterial adhesion and the mechanisms behind it are of crucial importance, and since this knowledge is limited, new information can hold the key to breakthroughs in either field.

Especially in the medical field where bacterial adhesion and the forthcoming biofilms cause life-threatening infections that are continuing to be more difficult to treat with antibiotics, detachment as well as adhesion prevention strategies are well sought after (Busscher et al. 2012; Campoccia et al. 2013; Swartjes et al. 2013; Swartjes et al. 2014a). To find out more about the mechanisms of bacterial adhesion, atomic force microscopy (AFM) has proven to be the tool of preference in order to determine the forces by which bacteria attach to surfaces and keep themselves adhered (Dufrene 2002; Dorobantu and Gray 2010; Müller and Dufrêne 2011; Dorobantu et al. 2012). The vertical motion of the AFM cantilever is often used to determine the force necessary to pull a bacterium from a cell or surface. This force, which has a magnitude of several nano Newton, is considered as the adhesion force (Dorobantu and Gray 2010). Whenever AFM is used to measure bacterial adhesion, the angle of the direction in which the bacterial adhesion force is measured and the substrate is approximately 90°. However, the amount of work (*W*) to overcome adhesion is a function of the pull-off angle *θ*, i.e., *W* = *F*·*d*·cos(*θ*), which indicates that the force required for detachment might change for different pull-off angles. Additionally, bacteria in most situations adhere from a flowing condition, in which the angle of approach leads to friction between a bacterium and the surface (Swartjes et al. 2014b). In fact, there is a relationship between adhesion and friction at nanoscale often used to describe the contact between two solid bodies, *F*_f_ = *µ* (*F*_n_ + *F*_adh_), stating that the friction force *F*_f_, equals the coefficient of friction (*µ*) multiplied by the sum of the normal force *F*_n_ and the adhesion force *F*_adh_ (Gao et al. 2004). In relation to these directional influences on bacterial adhesion, several methods have been applied to determine the lateral forces between bacteria and surfaces.

 A distinction can be made between two types of lateral forces; first, the shear adhesion force depending on the strength of the bond between an adhered bacterium and a surface, which breaks by moving the bacterium along the surface after it has adhered, and second, the lateral force arising between a bacterium and a surface when initial contact is made by a bacterium approaching the surface at an angle, representing the friction (Swartjes et al. 2014b). By challenging the shear strength of the adhesion bond using different flow rates of the liquid carrying the bacteria (Gazzola et al. 2015), or by detachment induced by passage of a liquid-air interface (Perera-Costa et al. 2014), estimations of the first type of lateral force have been made. However, since perpendicular to the surface the adhesion force of a single bacterium can be measured directly using the AFM, it is desirable to achieve a similar mode of action to measure the adhesion force at a different angle. Several attempts have recently been made to determine the lateral forces occurring between bacteria and surfaces using AFM (Verran et al. 2010; Zhang et al. 2011; Swartjes et al. 2014b). Quantification of the shear strength of bacterial adhesion has been achieved by imaging of bacteria; as the AFM cantilever moves along the surface in contact mode, the lateral movement of the cantilever can displace bacteria by pushing them away (Verran et al. 2010; Zhang et al. 2011). To measure lateral forces more directly, single-cell force spectroscopy (SCFS), in which a single bacterial cell is attached to the AFM cantilever, can be applied to probe the forces between this single bacterium and a surface. Kweon et al. modified an AFM cantilever with a bacterial spore and rubbed the spore against a silica surface to retrieve the values of occurring friction forces (Kweon et al. 2011). Even though this only involved a bacterial product, rather than an actual bacterium, the principle has also been performed using bacteria instead of a spore. The friction between polymer brush-modified surfaces and bacteria attached to a cantilever showed that friction was correlated to the amount of bacteria adhering to the surface, suggesting that friction forces play a role in attachment (Swartjes et al. 2014b). Interestingly, the friction and adhesion forces did not relate to each other as per the previously stated equation describing friction forces, indicating that bacterial friction and adhesion is more complex and challenging.

Most of these studies on lateral forces involved whole bacteria, however, based on the behavior of single proteins when subjected to forces at different angles, direction-dependent adhesion can also be studied by looking at components of bacterial adhesion complexes. SCFS has taken a flight over the last years and has expanded the insights about bacterial adhesion mechanisms considerably (Helenius et al. 2008; Müller and Dufrêne 2011; Isabey et al. 2013; Beaussart et al. 2014). Additionally, the technique has been extended to the use of single molecules, offering the possibility of isolating specific adhesion structures of bacteria and identifying their sole contribution in adhesion (Benoit et al. 2000; Sullan et al. 2015). Interestingly, single-molecule force spectroscopy (SMFS) using specific bacterial adhesion complexes reveals peaks in the force-distance curves due to breakage of multiple bonds, suggested to be caused by unfolding of the protein (Fig. 2C, D) and closely resembling the unzipping of previously mentioned proteins displaying anisotropic behavior (Fig. 2A, B) (El-Kirat-Chatel et al. 2014; Sullan et al. 2015). Additionally, measurements of a whole *Streptococcus mutans* cell suggest the presence of up to 10 ligand-receptor complexes being responsible for the binding of a single bacterial cell (Sullan et al. 2015).

**Fig. 2:**
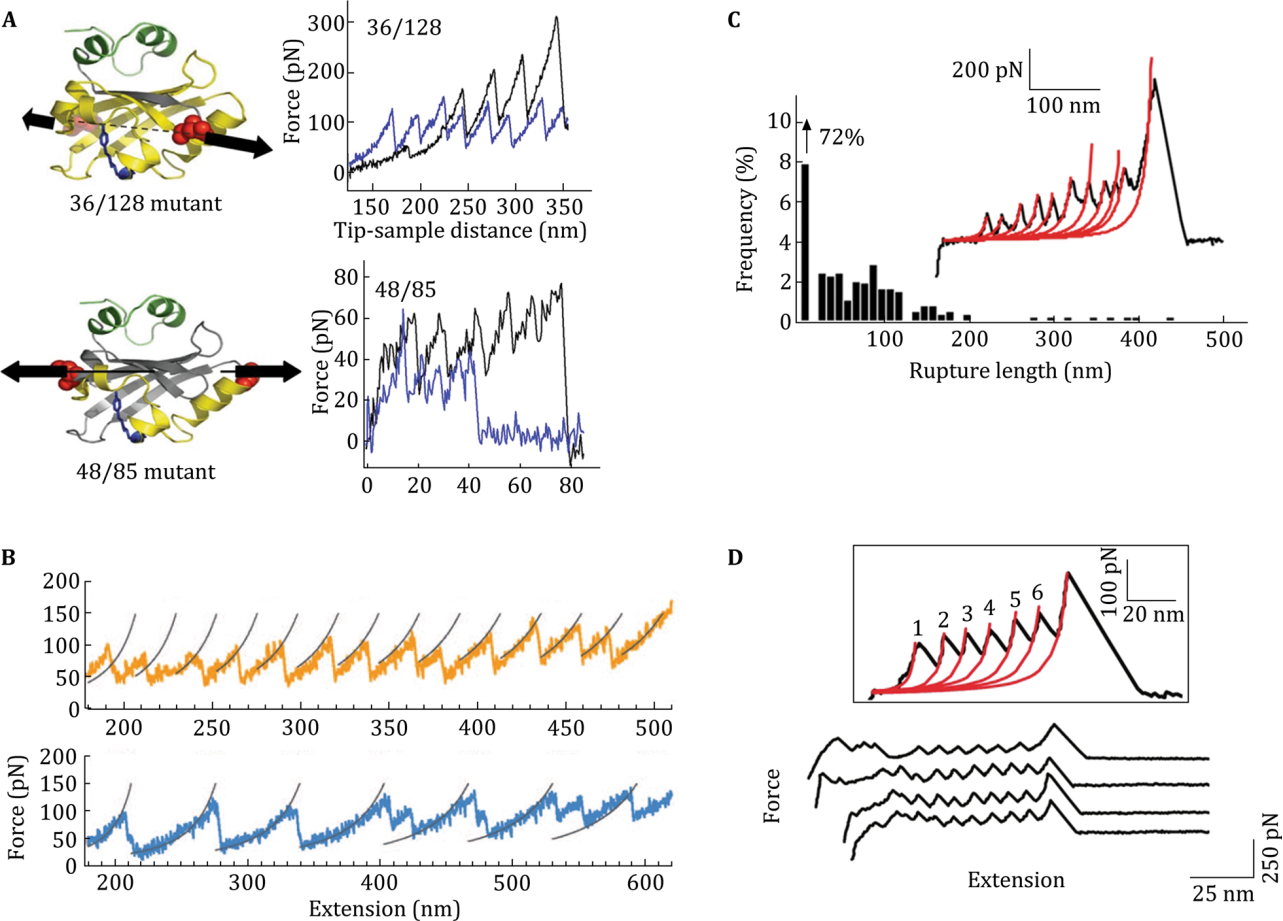
Unfolding behavior of proteins shown to have anisotropic responses to loading (**A**, **B**) and bacterial adhesion proteins displaying similar force patterns (**C**, **D**). **A** The distinct differences in force curves upon stretching of PYP by pulling at different axis. **B** Force-extension curves of unfolding of GFP displaying a distinct unzipping pattern for different directions of loading. **C** Force curves for the interaction between *S. mutans* adhesin P1 and fibronectin-coated solid substrates, exhibiting similar peaks observed for anisotropic proteins. **D** Unfolding force patterns of Als5p adhesion proteins closely resemble those of proteins known to respond differently to different loading directions. Adapted with permissions from Dietz et al. (2006), Nome et al. (2007), Alsteens et al. (2009) and Sullan et al. (2015)

Even though there are many examples showing that bond strength and adhesion phenomena in certain cases display properties highly depending on the direction of the applied force, direct measurements in the case of bacteria are scarce. In the quest for solutions to the bacterial adhesion problem, attention for shear and friction forces is present, as based on the previously mentioned examples and studies developing anti-fouling super-slippery surfaces (Wong et al. 2011; Epstein et al. 2012; Li et al. 2013; MacCallum et al. 2015), however, the fundamental role it plays in adhesion is not known. As the use of bacterial probes in AFM is increasing, unraveling the role of directionality could reveal completely new information on the adhesion mechanism of bacteria. Besides the added value it could have in non-specific adhesion, the example of a shear-dependent specific adhesion mechanism in *E. coli* suggests that specific interactions between ligands and receptors, which are present in the majority of bacteria, are able to exhibit different strengths based on how their unity is challenged. Given that specific patterns of unzipping and unfolding of individual proteins involved in bacterial adhesion are observed (Table 1), it is very well possible that the same directional dependent behavior seen in multiple types of proteins would also apply in the case of proteins associated with adhesion of bacteria. Additionally, for several pathogens it is suggested that zipper-like sequences are involved in host cell invasion, implicating that structures known to show directional dependent strength are partly responsible not only for general adhesion, but also for bacterial pathogenesis (Schwarz-Linek et al. 2003). Altogether, anisotropic adhesion behavior could not only stem from an array of adhesion complexes acting as individual bonds that can be loaded in a parallel or a zipper-like fashion, but also from within adhesion proteins or complexes where unfolding of single proteins might depend on the loading direction.

**Table 1:** Overview of different proteins whose behavior depends on the loading axis and of bacterial associated proteins whose unfolding characteristics imply the possibility of similar anisotropic behavior

Protein/structure	Interaction model	References
Ubiquitin	Direction-dependent unfolding	Carrion-Vazquez et al. (2003)
GCN4 protein	Anisotropic response to pulling	Gao et al. (2011)
Src SH3 protein	Anisotropic response to applied force	Jagannathan et al. (2012)
Green fluorescent protein (GFP)	Anisotropic deformation response	Dietz et al. (2006)
E2Lip3	Anisotropic unfolding	Brockwell et al. (2003)
Photoactive yellow protein (PYP)	Anisotropic unfolding	Nome et al. (2007)
*Escherichia coli* FimH	Shear-enhanced adhesion	Thomas et al. (2004), Aprikian et al. (2007), Yakovenko et al. (2008)
*Candida albicans* Als5p cell adhesion protein	Sequential unfolding	Alsteens et al. (2009)
*Bordetella pertussis* adhesin FHA	Sequential unfolding	Alsteens et al. (2013)
Pili from *Lactobacillus rhamnosus* GG	Zipper-like	Tripathi et al. (2013)
*Streptococcus mutans* P1 adhesin	Zipper-like	Sullan et al. (2015)
Als amyloids of *Candida albicans*	Zipper-like	Alsteens et al. (2012)

As shear is present almost everywhere inside the human body, e.g., in the oral cavity, blood vessels, intestine, and lungs, it suggests that firm attachment by resisting shear forces is mandatory for bacteria in order to persist in an adhered state. Proteins highly involved in the adhesion of different bacterial strains have shown to exhibit similar unfolding patterns compared to other proteins, which are highly anisotropic in their unfolding. Additionally, the shear strengthening of FimH in *E. coli* supports the implications for the possibility of direction-dependent adhesion mechanisms in bacteria, similar to those suggested for mammalian cells (Isabey et al. 2013). By probing friction forces and the adhesion forces lateral to the surface, specific information can be obtained that possibly provide new clues for anti-adhesive, or easy to clean surfaces. It is impossible to say which type of lateral force has more impact on bacterial adhesion, and the frictional forces probably contribute most to the transitions from unbound to surface attached, while it is likely that shear adhesion forces are most important in remaining an adhered state. As such, the frictional forces seem most interesting for design of non-fouling surface, while the shear adhesion force could help in designing strategies for bacterial removal. Nano-topographic surfaces could perhaps alter the direction in which bacteria experience shear forces, making them less likely to adhere, or easier to be removed.

Bacteria have outsmarted mankind by adapting resistance to a major part of our antibiotic spectrum, resulting in an increase in infections which are extremely hard to resolve (Spellberg et al. 2013; Wellington et al. 2013). In order to prevent infection there are many aspects of bacterial adhesion and biofilm formation requiring our utmost attention. The many suggestions for anisotropy of bacterial adhesion forces therefore imply that studying forces between bacteria and surfaces in multiple directions are desirable, as it might reveal precious information that can help in making crucial steps toward the development of new and more efficient anti-bacterial strategies.

## References

[CR1] Albrecht C, Blank K, Lalic-Mülthaler M, Hirler S, Mai T, Gilbert I, Schiffmann S, Bayer T, Clausen-Schaumann H, Gaub HE (2003). DNA: a programmable force sensor. Science.

[CR2] Alsteens D, Dupres V, Klotz SA, Gaur NK, Lipke PN, Dufrêne YF (2009). Unfolding individual Als5p adhesion proteins on live cells. ACS Nano.

[CR3] Alsteens D, Garcia MC, Lipke PN, Dufrêne YF (2010). Force-induced formation and propagation of adhesion nanodomains in living fungal cells. Proc Natl Acad Sci USA.

[CR4] Alsteens D, Ramsook CB, Lipke PN, Dufrêne YF (2012). Unzipping a functional microbial amyloid. ACS Nano.

[CR5] Alsteens D, Martinez N, Jamin M, Jacob-Dubuisson F (2013). Sequential unfolding of beta helical protein by single-molecule atomic force microscopy. PLoS One.

[CR6] Aprikian P, Tchesnokova V, Kidd B, Yakovenko O, Yarov-Yarovoy V, Trinchina E, Vogel V, Thomas W, Sokurenko E (2007). Interdomain interaction in the FimH adhesin of *Escherichia coli* regulates the affinity to mannose. J Biol Chem.

[CR7] Autumn K, Dittmore A, Santos D, Spenko M, Cutkosky M (2006). Frictional adhesion: a new angle on gecko attachment. J Exp Biol.

[CR8] Beaussart A, El-Kirat-Chatel S, Sullan RMA, Alsteens D, Herman P, Derclaye S, Dufrêne YF (2014). Quantifying the forces guiding microbial cell adhesion using single-cell force spectroscopy. Nat Protoc.

[CR9] Benoit M, Gabriel D, Gerisch G, Gaub HE (2000). Discrete interactions in cell adhesion measured by single-molecule force spectroscopy. Nat Cell Biol.

[CR10] Brockwell DJ, Paci E, Zinober RC, Beddard GS, Olmsted PD, Smith DA, Perham RN, Radford SE (2003). Pulling geometry defines the mechanical resistance of a beta-sheet protein. Nat Struct Biol.

[CR47] Busscher HJ, van der Mei HC, Subbiahdoss G, Jutte PC, van den Dungen JJAM, Zaat SAJ, Schultz MJ, Grainger DW (2012). Biomaterial-associated infection: locating the finish line in the race for the surface. Sci Transl Med.

[CR11] Campoccia D, Montanaro L, Arciola CR (2013). A review of the biomaterials technologies for infection-resistant surfaces. Biomaterials.

[CR12] Carrion-Vazquez M, Li H, Lu H, Marszalek PE, Oberhauser AF, Fernandez JM (2003). The mechanical stability of ubiquitin is linkage dependent. Nat Struct Biol.

[CR13] Cegelski L, Marshall GR, Eldridge GR, Hultgren SJ (2008). The biology and future prospects of antivirulence therapies. Nat Rev Microbiol.

[CR14] Dietz H, Berkemeier F, Bertz M, Rief M (2006). Anisotropic deformation response of single protein molecules. Proc Natl Acad Sci USA.

[CR15] Dorobantu LS, Gray MR (2010). Application of atomic force microscopy in bacterial research. Scanning.

[CR16] Dorobantu LS, Goss GG, Burrell RE (2012). Atomic force microscopy: a nanoscopic view of microbial cell surfaces. Micron.

[CR17] Dufrene YF (2002). Atomic force microscopy, a powerful tool in microbiology. J Bacteriol.

[CR18] Dunne WM (2002). Bacterial adhesion: seen any good biofilms lately?. Society.

[CR19] El-Kirat-Chatel S, Beaussart A, Boyd CD, O’Toole GA, Dufreîne YF (2014). Single-cell and single-molecule analysis deciphers the localization, adhesion, and mechanics of the biofilm adhesin LapA. ACS Chem Biol.

[CR20] Epstein AK, Wong TS, Belisle RA, Boggs EM, Aizenberg J (2012). Liquid-infused structured surfaces with exceptional anti-biofouling performance. Proc Natl Acad Sci USA.

[CR21] Foster TJ, Geoghegan JA, Ganesh VK, Höök M (2014). Adhesion, invasion and evasion: the many functions of the surface proteins of *Staphylococcus aureus*. Nat Rev Microbiol.

[CR22] Gao J, Luedtke WD, Gourdon D, Ruths M, Israelachvili JN, Landman U (2004). frictional forces and amontons’ law: from the molecular to the macroscopic scale. J Phys Chem B.

[CR23] Gao Y, Sirinakis G, Zhang Y (2011). Highly anisotropic stability and folding kinetics of a single coiled coil protein under mechanical tension. J Am Chem Soc.

[CR24] Gazzola G, Habimana O, Murphy CD, Casey E (2015). Comparison of biomass detachment from biofilms of two different *Pseudomonas* spp. under constant shear conditions. Biofouling.

[CR25] Helenius J, Heisenberg C-P, Gaub HE, Muller DJ (2008). Single-cell force spectroscopy. J Cell Sci.

[CR26] Hess H (2006). Self-assembly driven by molecular motors. Soft Mater.

[CR27] Isabey D, Féréol S, Caluch A, Fodil R, Louis B, Pelle G (2013). Force distribution on multiple bonds controls the kinetics of adhesion in stretched cells. J Biomech.

[CR28] Jagannathan B, Elms PJ, Bustamante C, Marqusee S (2012). Direct observation of a force-induced switch in the anisotropic mechanical unfolding pathway of a protein. Proc Natl Acad Sci USA.

[CR29] Kweon H, Yiacoumi S, Tsouris C (2011). Friction and adhesion forces of *Bacillus thuringiensis* spores on planar surfaces in atmospheric systems. Langmuir.

[CR30] Li J, Kleintschek T, Rieder A, Cheng Y, Baumbach T, Obst U, Schwartz T, Levkin PA (2013). Hydrophobic liquid-infused porous polymer surfaces for antibacterial applications. ACS Appl Mater Interfaces.

[CR31] Löfling J, Vimberg V, Battig P, Henriques-Normark B (2011). Cellular interactions by LPxTG-anchored pneumococcal adhesins and their streptococcal homologues. Cell Microbiol.

[CR32] MacCallum N, Howell C, Kim P, Sun D, Friedlander R, Ranisau J, Ahanotu O, Lin JJ, Vena A, Hatton B, Wong TS, Aizenberg J (2015). Liquid-infused silicone as a biofouling-free medical material. ACS Biomater Sci Eng.

[CR33] Matouschek A, Bustamante C (2003). Finding a protein’ s Achilles heel. Nat Struct Biol.

[CR34] Müller DJ, Dufrêne YF (2011). Atomic force microscopy: a nanoscopic window on the cell surface. Trends Cell Biol.

[CR35] Nome RA, Zhao JM, Hoff WD, Scherer NF (2007). Axis-dependent anisotropy in protein unfolding from integrated nonequilibrium single-molecule experiments, analysis, and simulation. Proc Natl Acad Sci USA.

[CR36] O’Toole GA, Kaplan HB, Kolter R (2000). Biofilm formation as microbial development. Annu Rev Microbiol.

[CR37] Perera-Costa D, Bruque JM, González-Martín ML, Gómez-García AC, Vadillo-Rodríguez V (2014). Studying the influence of surface topography on bacterial adhesion using spatially organized microtopographic surface patterns. Langmuir.

[CR38] Schwarz-Linek U, Werner JM, Pickford AR, Gurusiddappa S, Kim JH, Pilka ES, Briggs JAG, Gough TS, Höök M, Campbell ID, Potts JR (2003). Pathogenic bacteria attach to human fibronectin through a tandem beta-zipper. Nature.

[CR39] Spellberg B, Bartlett JG, Gilbert DN (2013). The future of antibiotics and resistance. N Engl J Med.

[CR40] Sullan RMA, Li JK, Crowley PJ, Brady LJ, Dufrêne YF (2015). Binding forces of *Streptococcus mutans* P1 Adhesin. ACS Nano.

[CR41] Swartjes JJTM, Das T, Sharifi S, Subbiahdoss G, Sharma PK, Krom BP, Busscher HJ, Van der Mei HC (2013). A functional DNase I coating to prevent adhesion of bacteria and the formation of biofilm. Adv Funct Mater.

[CR42] Swartjes JJTM, Sharma PK, van Kooten T, Van der Mei HC, Mahmoudi M, Busscher HJ, Rochford ETJ (2014). Current developments in antimicrobial surface coatings for biomedical applications. Curr Med Chem.

[CR43] Swartjes JJTM, Veeregowda DH, Van der Mei HC, Busscher HJ, Sharma PK (2014). Normally oriented adhesion versus friction forces in bacterial adhesion to polymer-brush functionalized surfaces under fluid flow. Adv Funct Mater.

[CR44] Thomas WE, Nilsson LM, Forero M, Sokurenko EV, Vogel V (2004). Shear-dependent ‘stick-and-roll’ adhesion of type 1 fimbriated *Escherichia coli*. Mol Microbiol.

[CR45] Tian Y, Pesika N, Zeng H, Rosenberg K, Zhao B, McGuiggan P, Autumn K, Israelachvili J (2006). Adhesion and friction in gecko toe attachment and detachment. Proc Natl Acad Sci USA.

[CR46] Tripathi P, Beaussart A, Alsteens D, Dupres V, Claes I, Von Ossowski I, De Vos WM, Palva A, Lebeer S, Vanderleyden J, Dufreîne YF (2013). Adhesion and nanomechanics of pili from the probiotic *Lactobacillus rhamnosus* GG. ACS Nano.

[CR48] Verran J, Packer A, Kelly PJ, Whitehead KA (2010). Use of the atomic force microscope to determine the strength of bacterial attachment to grooved surface features. J Adhes Sci Technol.

[CR49] Vlamakis H, Chai Y, Beauregard P, Losick R, Kolter R (2013). Sticking together: building a biofilm the *Bacillus subtilis* way. Nat Rev Microbiol.

[CR50] Wellington EMH, Boxall ABA, Cross P, Feil EJ, Gaze WH, Hawkey PM, Johnson-Rollings AS, Jones DL, Lee NM, Otten W, Thomas CM, Williams AP (2013). The role of the natural environment in the emergence of antibiotic resistance in Gram-negative bacteria. Lancet Infect Dis.

[CR51] Wong TS, Kang SH, Tang SKY, Smythe EJ, Hatton BD, Grinthal A, Aizenberg J (2011). Bioinspired self-repairing slippery surfaces with pressure-stable omniphobicity. Nature.

[CR52] Yakovenko O, Sharma S, Forero M, Tchesnokova V, Aprikian P, Kidd B, Mach A, Vogel V, Sokurenko E, Thomas WE (2008). FimH forms catch bonds that are enhanced by mechanical force due to allosteric regulation. J Biol Chem.

[CR53] Zhang T, Chao Y, Shih K, Li XY, Fang HHP (2011). Quantification of the lateral detachment force for bacterial cells using atomic force microscope and centrifugation. Ultramicroscopy.

